# Very Late-Onset Serious Chronic Adverse Effects After Radical Chemoradiotherapy for Anal Canal Cancer

**DOI:** 10.3390/jcm14113841

**Published:** 2025-05-29

**Authors:** Pavel Vitek, Jiri Kubes, Barbora Ondrova, Alexandra Haas

**Affiliations:** Proton Therapy Center Czech, 180 00 Prague, Czech Republic; jiri.kubes@ptc.cz (J.K.); barbora.ondrova@ptc.cz (B.O.); alexandra.haas@ptc.cz (A.H.)

**Keywords:** anal cancer, radical radiotherapy, electron boost, fibroatrophy, osteoradionecrosis, hyperbaric oxygen, late effects

## Abstract

Radical chemoradiotherapy has been used as a frontline treatment for squamous cell cancer of the anus for the last 30–40 years. Considerable acute and chronic adverse effects have been observed following radiotherapy using 2D and 3D techniques. A case of very late-onset severe chronic toxicity in a patient 26 years after radiotherapy is presented. The patient underwent radical chemoradiotherapy for squamous anal cancer stage T3N3M0 in 1998. In the anal region, cumulative doses up to 77.6 Gy (including electron boost) were administered. Durable complete regression of the disease was achieved. Fourteen years after treatment, the patient developed vast fibroatrophy of the anus and perineum, progressing within the subsequent four years to necrosis and sphincter loss. Twenty years after treatment, the asymptomatic osteonecrotic foci in the left femur appeared on MRI scans. Despite two courses of hyperbaric oxygen treatment, the fibroatrophy and subsequent necrosis of soft tissues remained progressive, but the osteonecrosis was stable. Twenty-six years after treatment, the progressive changes induced symptomatic osteomyelitis of the ischium and pubic bone. The patient now requires permanent supportive treatment. The presented case is exceptional in the very late-onset typical chronic adverse effects developing after non-conformal radiotherapy administered at high doses as part of contemporary treatment protocols. There is little evidence regarding the late onset of chronic adverse effects, since the follow-up period is usually shorter than that of the case presented. Moreover, a significant portion of patients do not survive to reach the late-onset period of adverse effects. The presented case shows that there may be long-term survivors of anal cancer in the population who were treated with outdated techniques and who still carry a risk of late-onset severe, progressive adverse effects.

## 1. Introduction

Chemoradiotherapy became a standard first-line treatment for anal cancer due to its ability to preserve the anal sphincter and favourable local control data obtained in phase III trials [1,2,3].

There is a sufficient body of data on acute toxicity coming from clinical trials; however, data on late toxicity are scarce due to later onset of complications frequently exceeding the length of follow-up periods in trials. Incidence data are available for gastrointestinal late effects—7.0–64.5% [4,5,6,7,8]. There are few data indicating the incidence of radiation dermatitis and fibrosis at 8.8–9.2% [8,9]. The incidence of more advanced effects—radiation-induced fibroatrophy (RIF) and necrosis—remains unknown. Hip osteoradionecrosis (ORN) has been identified as a frequent complication of older radiotherapy techniques used to treat pelvic cancer, and anal cancer has contributed up to 14.8% of cases [10].

RIF and ORN are considered significant late adverse effects with substantial functional consequences. Their pathophysiology is described in detail elsewhere [11,12,13]. The usual onset period is from 6 months to 4–5 years after radiotherapy [10,14]. Onset periods longer than 10 years after radiotherapy of anal cancer have not been referred. It is important to note that the median observation period in the referred groups is shorter than 10 years. A large population-based study (1985–2000) revealed a 5-year survival rate of 51.8% [15]. However, references coming from later periods and smaller cohorts indicate more favourable 5-year survival rates of 68.9–83.6% [16,17].

Dosimetry—total and integral doses of radiation in healthy tissues are highly predictive of late toxicity. Intensity-modulated radiotherapy (IMRT) trials have shown significant decreases in the incidence of any late toxicity compared to older 2D and 3D techniques [18,19]. Two retrospective studies document a significant decrease in late toxicity in patients who received a dose lower than 60 Gy [20,21]. Significant reductions in integral doses have been documented for particle therapy [22,23], and a reduction in late toxicity may be expected; however, a representative analysis is not yet available.

There are limited treatment options for both RIF and ORN. A modest antifibrotic effect has been revealed for anti-inflammatory agents and pentoxifylline with antioxidant vitamin E [24,25]. Other agents—TGF-b1 inhibitors, tyrosine kinase inhibitors, and PI3K/AKT/mTOR inhibitors—are being investigated in preclinical and clinical trials [26,27,28,29,30,31]. Hyperbaric oxygen has shown improvements in physical functions and general health status in nine patients with radiation-induced anal fibrosis and/or ulceration [32,33]. Prevention—via a reduction of integral doses administered to skin, soft tissues, and bones—remains the best and most effective way to cope with adverse effects.

A single case of very late-onset, slowly progressive RIF and ORN with considerable functional consequences is presented.

## 2. Case History

The patient, a 24-year-old male, was diagnosed with anal canal cancer stage T3N3M0 (UICC 7th edition) in 1998. There was an exophytic voluminous tumour at the ventral part of the anal verge, invading the perineum, sized 6–7 cm in longitudinal direction. The inguinal nodes were bilaterally enlarged, sized up to 20 mm, classified as metastatic. Biopsy of the primary lesion confirmed squamous cell cancer coded M8070/3. Pelvic and abdominal CT scans excluded any other metastatic lesions. MR imaging was not available at the time of diagnosis.

Radical chemoradiotherapy was indicated as part of the contemporary protocol at the Institute of radiation oncology, Prague, CZ. Two-dimensional planning was based on CT imaging. Two-dimensional treatment was administered at linear accelerators: X-rays 5 MeV, electrons 15 MeV (Orion 5, Saturne 18, CGR MeV, F-78530 Buc, CGR MeV, Paris, France). Doses in organs at risk were not separately assessed.

Treatment volume consisted of the following three shrinking CTVs.

**Figure 1:**
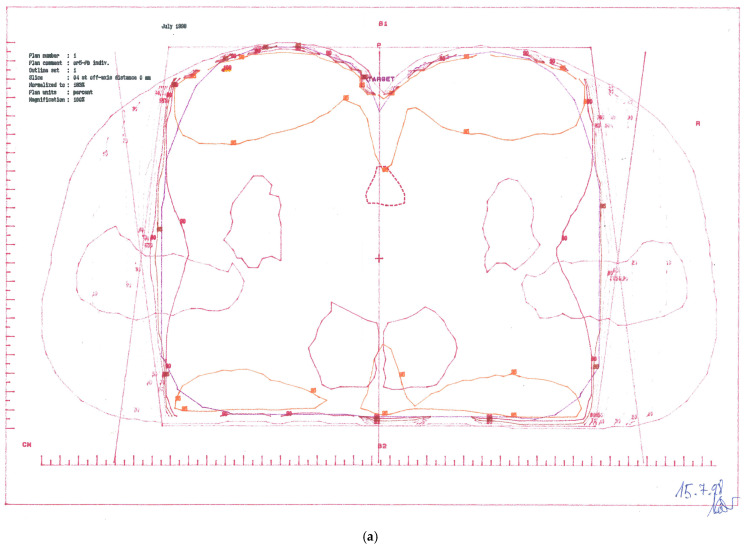

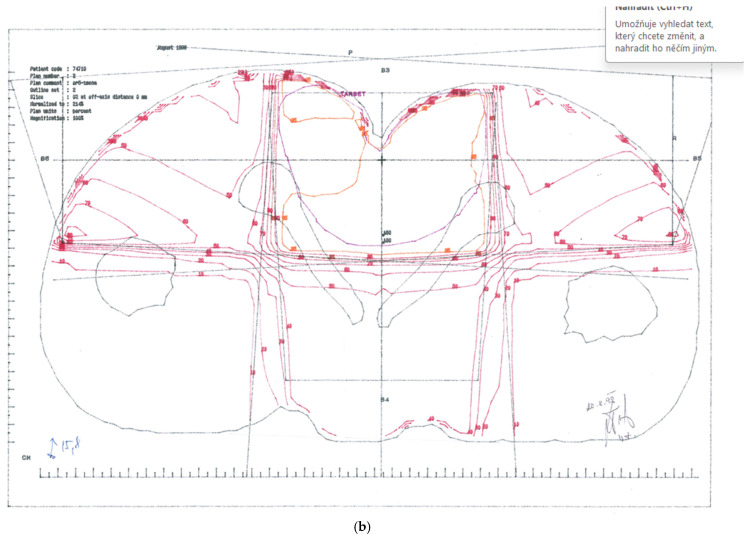
Treatment plans. (**a**) CTV-1 encompassing anus, perianal skin, whole pelvis, and groins (step 1). (**b**) CTV-2 encompassing anus, perianal skin, and dorsal pelvis (step 2).

CTV-1: Primary tumour and perianal skin, whole pelvis, and groins—cumulative dose 57.6 Gy; (Figure 1a)

CTV-2: Primary tumour and perianal skin, as well as dorsal pelvis—cumulative dose 67.6 Gy; (Figure 1b)

CTV-3: Primary tumour and perianal skin—cumulative dose 77.6 Gy.

A rather complicated sequential treatment regimen was planned in three steps using three different techniques, as shown in Table 1. The treatment period lasted from 15-JUL-1998 to 03-SEP-1998, with a total treatment time of 51 days (34 treatment days).

Concomitant chemotherapy was administered within the contemporary treatment protocol below.

- Mitomycin C 20 mg, intravenous injection, day 1;
- 5-fluoruracil 750 mg/24 h, continuous intravenous infusion, days 1–4 and 29–32.

The treatment was administered according to the prescribed plan without any interruptions. Acute toxicity was mild, not exceeding grade 2.

The effect was assessed by means of digital rectal examination (DRE) 2 months after radiotherapy. Residual induration remained on the ventral side of the anus. Fine-needle aspiration biopsy showed fibroproductive inflammatory changes, but no residual cancer cells. A CT scan 2 months after radiotherapy confirmed complete regression of the primary tumour, no regional lymphadenopathy, and no distant metastases.

One year after radiotherapy, DRE showed only small fibrotic foci about 2 cm in size on the ventral side of the anal verge. A CT scan showed no pathologic changes in the pelvis and no lymphadenopathy. There were no functional changes. The sphincter was fully functional, and defecation reflexes were fully restored.

The patient attended follow-up examinations—DRE annually and CT scan biannually for the next 11 years. There were no signs of late toxicity within 11 consecutive years after radiotherapy. Good sphincter function was maintained, and there was no radiation proctitis, no perianal fibrosis, and no skeletal abnormalities within the pelvic and femoral bones. The patient’s condition was good, and he was not limited in his daily activities.

Twelve years after radiotherapy, the patient presented with progressive perianal induration, sphincter dysfunction, and temporary limb swelling. A CT scan still showed no relapse and no lymphadenopathy.

Fourteen years after radiotherapy, the patient suffered from a subjective symptom of “painless disruption” at the perineum. From a basic physical exam, there was apparent induration of the perianal region and anal sphincter, while the rugged surface of the perianal skin resembled local relapse (an appearance very similar to skin cancer). The patient underwent multifocal biopsy of the skin; however, it did not reveal any signs of cancer, only signs of advanced skin and subdermal fibrosis. An MRI scan showed signs of perianal and periproctal fibrosis. This finding did not change for 4 years.

Eighteen years after radiotherapy, deep excavation at the site of the perineum appeared, with a rugged bottom again resembling tumour relapse. Multiple biopsies again indicated fibrosis and no cancer cells.

Nineteen years after radiotherapy, MRI had become available at the follow-up facility and the patient underwent periodic imaging. Twenty years after radiotherapy, MRI showed new skeletal changes—osteonecrosis of the right femoral head, right acetabulum, and femoral diaphysis. However, there were no subjective symptoms. Progressive sphincter dysfunction became disturbing and resulted in colostomy.

Twenty years after radiotherapy, the patient commenced hyperbaric oxygen (HBO) therapy in a hyperbaric chamber, at a pressure of 1.5 bar, breathing 100% oxygen through a mask. The patient underwent three consecutive cycles of HBO within 10 months, each cycle containing 20 sessions of 90 min, with the cycles lasting 80 days, 120 days, and 90 days. The total treatment time was 10 months.

HBO therapy had no impact on the local lesion, and the patient did not report any functional improvements.

Excavation at the site of the perineum slowly increased in size up to the 23rd year after radiation, and it completely depleted the sphincter. Then it remained stable for the next 3 years. The patient did not receive any specific therapy except for temporary antibiotic retreatment to resolve remittent wound infections in the area of fibroatrophy and necrosis.

**Figure 2:**
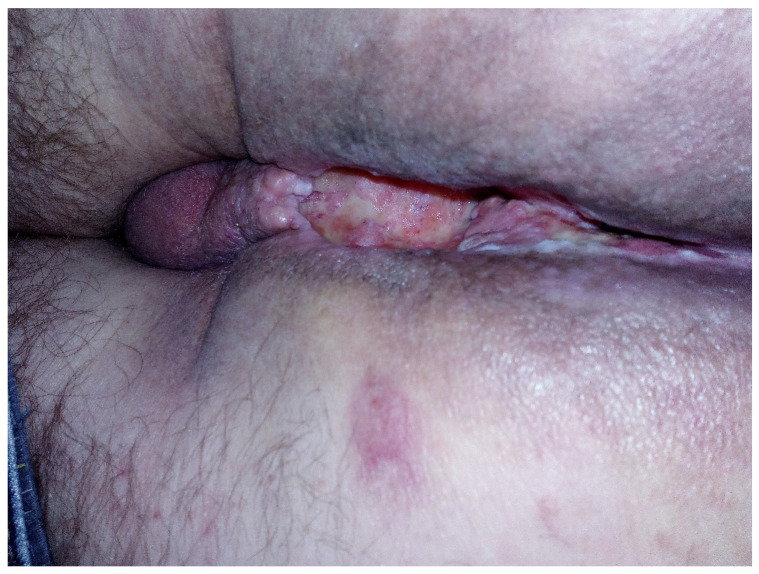
Perineal excavation involving the region of the anal canal.

**Figure 3:**
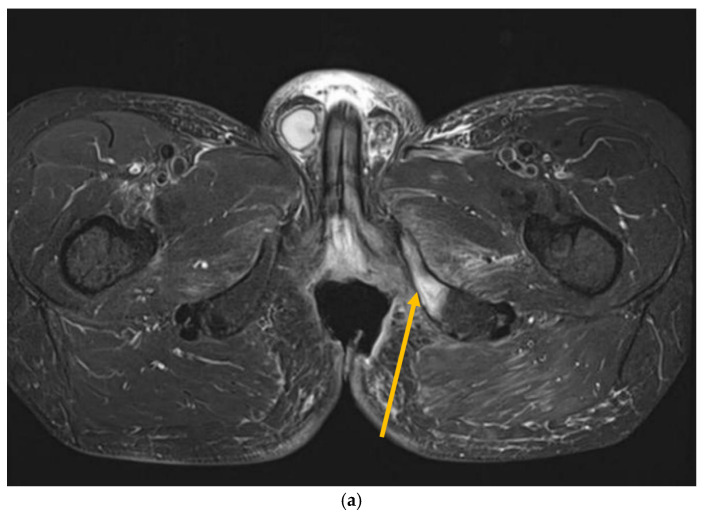

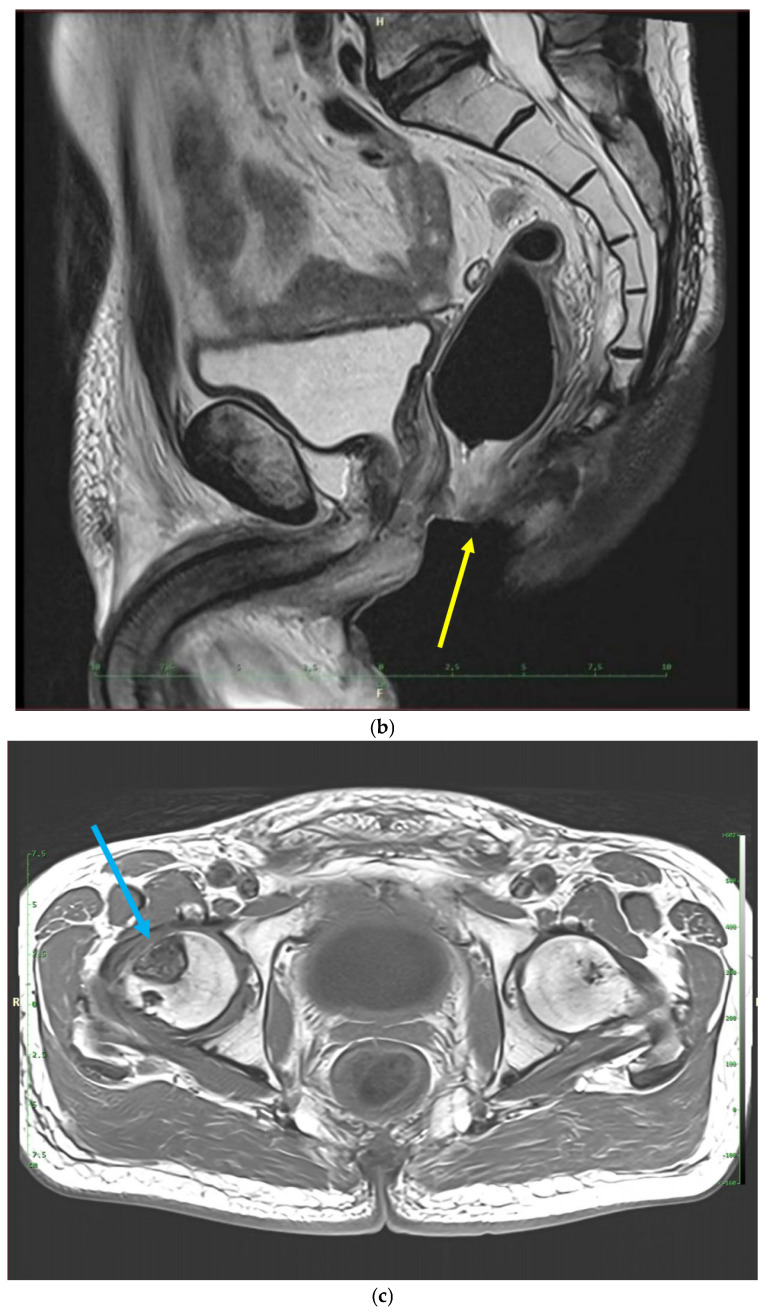
MRI scans 26 years after radiotherapy: T1-weighted axial MRI scan shows vast perineal and anal defects progressing to the left lower arm of the pubis with signs of induced osteomyelitis (orange arrow) (**a**); T2-weighted sagittal scan shows vast perineal and anal defects including absence of anal sphincter (yellow arrow) (**b**); and T1-tse weighted axial scans show the osteoradionecrosis of the right hip (blue arrow) (**c**).

Recently, inflammatory reactions adjacent to the necrosis have slowly progressed to the lower arm of the left pubic and ischium bone and have induced mild osteomyelitis demanding antibiotic and analgesic treatment (Figure 2 and Figure 3). The osteonecrotic focus within the right femoral head and acetabulum is now stable, asymptomatic, and painless.

The patient is now 26 years post-radiotherapy. He is naturally limited by his irreversible colostomy and weak mucinous rectal secretions, which are unpredictably released as a result of the absent sphincter. The recently developed osteomyelitis is a substantial complication limiting the patient in his job—he is a driver of agriculture machines. The patient experiences negligible limitations resulting from the osteoradionecrosis of his right hip.

## 3. Discussion

Chronic adverse effects of radiotherapy including radiation-induced fibroatrophy (RIF) and osteoradionecrosis (ORN) may be expected and have been consistently described [10,14,34]. RIF after radiotherapy for anal cancer may proceed to necrosis and fistulation, followed by various functional consequences. Sphincter dysfunction and faecal incontinence have been predominantly described [8,35,36,37]. Various fistulations have been reported in 0.7–22% of cases and faecal incontinence in 0.5–10.9% [38,39]. Abdominoperineal resection, or at least derivation colostomy, follows frequently. The colostomy rate resulting from chronic adverse effects is estimated at 1.5–12% [7,20,40,41,42] (Table 2). The colostomy rate resulting from local relapse (either as part of salvage surgery—abdominoperineal resection—or as a separate derivation colostomy only) is about 3 times higher than that resulting from toxicity, i.e., 9–22% vs. 0–6.3% [7,20,40,41,42,43,44] (Table 2).

The onset of fibrosis and its sequelae is usually described much earlier than in this case, some 4–12 months after radiotherapy [43,45]. However, the median follow-up times in the referred anal cancer patient groups (22–100 months) may not be long enough to provide reproducible information on late- or very late-onset chronic toxicity [7,20,40,41,42] (Table 2).

In general, there are only a few references describing very late-onset chronic adverse effects. Different localizations, fractionation schedules, treatment volumes, and organs are concerned [46]. Johansson et al. presented long observation periods after chest wall radiotherapy for breast cancer, up to 30 years [47]. RF was shown to develop in the first decade, not later. Other effects like brachial plexus neuropathy and paralysis were observed later, even after 30 years. Moreover, there is the question of applicability. Any observation study presented 20 years or more after the treatment period is naturally based on rather obsolete techniques of radiotherapy and obsolete fractionation regimens. It is questionable, then, to what degree the results are applicable to current treatments beyond simple awareness of particular adverse effects.

The median period to ORN onset is less than 4 years after radiotherapy [10]. However, very long periods have also been observed for jaw ORN—up to 38 and 45 years after radiotherapy [48,49]. ORN has more frequently been described in relation to older techniques of radiotherapy predominantly involving the jaw. The hip presents as the second most frequent region at risk of ORN. Again, it is related to extensive irradiation with outdated techniques used for pelvic radiation, e.g., the “box” technique. Anal cancer radiotherapy comprised 14.8% of hip ORN cases in a survey covering the period of 1980–2020 [10].

The presented case shows a very late onset of two chronic adverse effects: skin and sphincter fibroatrophy/necrosis and osteoradionecrosis. These effects are rather exceptional in terms of delayed onset. Fibrosis slowly proceeded to fibroatrophy and finally necrosis, with consequent anatomic and functional impairment. Single successive events that are relevant to specific stages of RIF have been described in detail by Delanian et al. [45]. The final effect has presented as continuously and slowly increasing vast tissue defects—complete sphincter disintegration and loss, both anatomic and functional in this case. In contrast to the observed fibroatrophy, the exceptionally delayed onset of unilateral hip ORN has remained stable and asymptomatic and has not developed any functional disturbances despite no consistent treatment except hyperbaric oxygen.

Both adverse effects are apparently related to the radiotherapy technique. A full dose was homogeneously administered to the entire pelvis via the “box” technique, while the prescribed dose was maximal in terms of contemporary recommendations and references [50,51]. Moreover, the electron boost covered the subcutaneous tissue and skin adjacent to the primary tumour in the range relevant to current tissue loss and disintegration. A lower dose administered to the pelvic bone (outside the volume of the 10 Gy boost dose) may have contributed to the different behaviour of soft tissue (continuously progressive fibroatrophy and necrosis) and bone (no progression of necrosis for the last 8 years).

The severe chronic adverse effects, at least the perineal and sphincter fibrosis, may have been expected if the total dose was considered for the combined hyper and normofractionation regimen. But the late onset of severe adverse effects was not predictable. However, there may be some bias considering what is the “usual” onset time of these adverse effects. Patient groups with a consistent follow-up regime and reproducible data present rather short median follow-up times (Table 1). Late-onset cases are referred to as “exceptional” since they are presumably usually excluded from references and observations. Moreover, for anal cancer, the 5-year survival rates are referred between 65% and 85% [42,52,53], and, although limited, the data for 10-year survival reveal a rate up to 75% [54]. A large population-based study (1985–2000) indicated the 5-year survival rate as only 51.8% [15]. Survival data may produce some bias in estimating the true risk of chronic adverse effects with a very late onset.

The treatment options for developed fibrosis and fibroatrophy are limited. Favourable results have been achieved with anti-inflammatory agents or pentoxifylline combined with the antioxidant tocopherol [24,25]. Overall, the effects are classified as “modest”. However, the treatment was not prescribed in the referred case. Other agents—TGF-b1 inhibitors, tyrosine kinase inhibitors, and PI3K/AKT/mTOR inhibitors—are being investigated in preclinical and clinical trials; treatment is not available for a routine practice. HBO is another available option [32,33,55]. Its efficacy has been assessed predominantly in terms of improvements in functional deficits, not observed in the referred case. HBO remained without significant effects. However, in the stage of developed fibroatrophy and necrosis, any significant improvement can hardly be expected with any method, and treatment can only be employed with the aim to preclude further progression. Sphincter loss and functional impairment can be compensated only by colostomy with or without abdominoperineal resection. Any functional restoration can also be hardly expected. There is a more effective option for osteoradionecrosis compensation using total hip joint endoprosthesis. However, the asymptomatic course and lack of progression still allow us to postpone surgery in this case.

Finally, the presented case stresses the importance of prevention—more favourable dosimetry. It confirms the significance of reasonable dosage and the use of more advanced technologies, such as high-voltage radiation (IMRT, VMAT) or particle therapy, to achieve higher conformity and lower doses outside the target volume. Looking to the future, fewer chronic adverse effects may be expected, including late-onset effects. However, there are still plenty of long-term survivors undergoing follow-up, who were treated some decades ago by obsolete techniques and with higher doses, the distribution of which may not be reproducible. These patients carry a “heritage” of unfavourable dosimetry and remain at risk of late-onset severe adverse effects. 

## 4. Conclusions

The late adverse effects of radiotherapy may keep developing even decades after treatment within the framework of a very complicated pathophysiology, finally resulting in substantial functional and anatomic impairment. The treatment options are very limited. The risk of these adverse effects should be considered in long-term survivors after radiotherapy administered with outdated techniques and unfavourable dosimetry. Mutilating complications are demonstrated in the presented case of anal canal cancer; nevertheless, survivors of various other malignancies may also be similarly affected.

## Figures and Tables

**Table 1:** Volumes and dosage.

Step	Dose per Fraction	Number of Fractions	Volume	Technique	Dose	Cumulative Dose
1	1.2 Gy hyperfractionation 2 fractions/day	48 (10 fractions/week)	anus, perianal skin, whole pelvis, and groins (CTV 1)	X-rays 5 MeV Isocentric 2 opposed fields (dorsal, ventral)	57.6 Gy	57.6 Gy
2	2 Gy normofractionation	5 (5 fractions/week)	anus, perianal skin, and dorsal pelvis (CTV 2)	X-rays 5 MeV Isocentric 4 fields (“box”)	10 Gy	67.6 Gy
3	2 Gy normofractionation	5 (5 fractions/week)	boost: anus perianal skin (CTV 3)	Electrons 15 MeV SSD 1 direct field	10 Gy	77.6 Gy

**Table 2:** Late effects and colostomy results described in selected studies on anal cancer.

Study, Reference	Number of Patients	Median Follow-Up Time	Late Effects	Incidence (Grading)	Colostomy Rate, Reason		Rem
					Relapse	Adverse Effects	
Gerard et al. [43]	95	64 mo, (18 pts. > 8 years)	Anal necrosis	Any grade 14/95 (14.7%)	11/95 (11.6%)	6/95 (6.3%)	Median onset time 11 months (6–18) 18 pts. follow-up > 8 years
De Bari et al. [7]	120	65 mo (4–238)	Anorectal	G3 10/120 (7.5%) G4 1/120 (0.8%)	16/122 (13.1%)	2/120 (1.7%)	
Ortholan et al. [20]	66	66 mo	Bleeding Anal fibrosis Ulceration	G1-G2 10/66 (15.2%) G3 3—1/66 (1.5%) G1-G2—2/66 (3.0%) G1-G2—6/66 (9.1%)	5/66 (7.5%)	1/66 (1.5%)	
UNICANCER ACCORD 03 trial Pfeiffert et al. [40]	283	50 mo (0–102)	Ulceration/fistulation Bleeding Diarrhoea Incontinence	G3 21/283 (7.4%) G4 16/283 (5.6%) G3 77/283 (27.2%) G4 1/283 (0.4%) G3 10/283 (4%) G4 4/283 (1.4%) G3 35/283 (12.4%) G4 10/283 (4%)	43/283 (15.2%)	9/283 (3.2%)	
Young et al. [41]	120	21.6 mo (1–241)	Incontinence Bleeding Stenosis Telangiectasia	G1-G2 15/120 (12.5%) G1-G2 11/120 (9.2%) G1-G2 2/120 (1.7%) G1-G2 8/120 (6.7%)	7/120 (5.8%)	0/120 (0%)	No grade 3–4 late effects Median dose 39.6 Gy
Swedish national ANCA cohort study Johansson et al. [42]	388	85 mo (0–113)	n.a.	n.a.	n.a.	n.a.	Retrospective Swedish Cancer Registry-based study
Madhu J. et al. [44]	34	98 mo (8–169)	Skin ulceration Anal canal ulceration	G3–4 3/34 (8.8%) G3 1/34 (2.9%)	0/34 (0%)	0/34 (0%)	

## Data Availability

The original contributions presented in this study are included in the article. Further inquiries can be directed to the corresponding author.

## Abbreviations

The following abbreviations are used in this manuscript.

CT	Computer tomography
CTV	Clinical target volume
DRE	Digital rectal examination
HBO	Hyperbaric oxygen
IMRT	Intensity-modulated radiation therapy
ORN	Osteoradionecrosis
MRI	Magnetic resonance imaging
RIF	Radiation-induced fibroatrophy
VMAT	Volumetric modulated arch therapy
